# Clinical Memoranda from the Surgical Clinic at the Sisters’ of Charity Hospital

**Published:** 1894-01

**Authors:** Herman Mynter

**Affiliations:** Professor of Surgery, Niagara University, and Surgeon to the Sisters’ Hospital


### CLINICAL MEMORANDA FROM THE SURGICAL CLINIC AT THE SISTERS OF CHARITY HOSPITAL.

By HERMAN MYNTER, M. D.,
Professor of Surgery, Niagara University, and Surgeon to the Sisters' Hospital.

### FRACTURES AT THE ELBOW-JOINT.

Fractures at or near the elbow-joint are of frequent occurrence, particularly in childhood, and apt to be followed by more or less deformity and anchylosis. The prognosis is always somewhat doubtful, and, as Gerster says, "a very guarded prognosis is, on the part of the physician, a sign of wisdom and discretion." How to prevent the deformity and anchylosis is, for all, the important point. The deformity consists, when there is no dislocation present, in the loss of "the carrying function," by which we understand the normal obtuse angle in the elbow-joint, which keeps the forearm in abduction when extended, and thus enables a person to carry weights without interfering with locomotion. This obtuse angle in extension is produced by the trochlea extending about one-quarter inch farther downward than the external condyle or capitulum, with which the radius articulates, so that the ulna moves on a larger circle than the radius, and a line tangent on the lower end of the trochlea and capitulum forms an obtuse angle inward with a longitudinal line through the humerus. The obtuse angle disappears in flexion, and the forearm and humerus become parallel. In fractures through the internal condyle, implicating the whole trochlea, or in T fractures, the fragment with the ulna attached will slide upward and generally backward, on account of the obliquity of the line of fracture, this running, usually, downward and forward. This sliding upward is mainly produced by muscular contractions, probably, of the triceps and brachialis anticus. The trochlea and capitulum come on the same level, and the result will be obliteration of the obtuse angle, *i. e.*, adduction of the forearm in extension, or loss of the carrying function. The same may happen in supracondylar fractures. Stimson states that the ascent of the internal condyle of one-quarter of an inch will destroy the normal obtuse angle at the elbow. The upper end of the lower fragment is very apt to be tilted forward by the contraction of the triceps, and, if union takes place in this position, hyperextension in the elbow with impaired flexion may result.

Partial dislocation may complicate and obscure the fracture.

While the deformity is mostly dependent upon the loss of the carrying function, the partial anchylosis, with impairment of flexion, is the result particularly of the tilting forward of the lower fragment, or overlapping of the upper fragment, or an abnormally developed callus, or rotation forward of the articulating surfaces of the fragmina. In either case, the coronoid process of the ulna or the radius may meet a bony prominence in the plica cubiti, which will prevent flexion, and no amount of force will overcome the anchylosis.

In regard to treatment, there are, as Packard states, four difficulties to overcome: (1) To keep the fragments in contact; (2) to prevent the formation of an angle salient forwards, *i. e.*, hyperextension; (3) to maintain the oblique line of the articulation by avoiding upward pressure on the inner portion of the joint-surface of the lower fragment; (4) and to obviate stiffening of the elbow.

Still another difficulty is to prevent, in T fractures, gaping of the fractured portions above and the insertion between them of part of the upper fragment, thus increasing the lateral distance between the condyles and broadening the epiphysis.

In regard to treatment, surgeons are divided into two camps: those who advocate treatment with the arm extended, and somewhat abducted, for fear of losing the carrying function; and those who advocate treatment with the arm flexed to a right angle, for fear of anchylosis. The first camp deprecate early passive motions, and make light of the danger of anchylosis, pointing to the fact that we, as orthopedic surgeons have shown, can keep joints immobilized for a great length of time without danger of anchylosis. While this is true as far as healthy joints are concerned, it is wrong when we have to do with joint fractures with intra-articular bleeding. Blood may organize and adhesions form from this source alone, but the synovial membrane itself is apt to take part in the process through a traumatic arthritis, form granulation tissue and secondarily strong adhesions.

Dr. Jarvis Wright reports, in *Annals of Surgery*, August, 1893, ten cases of anchylosis in the elbow-joint, following treatment with the arm extended, a sufficient proof that this treatment is not without danger. If such an accident should occur, the arm is useless for all practical purposes, and operative interference, *i. e.*, resection or arthrotomy, becomes necessary.

If passive motions be not made, the danger of anchylosis is increased. The second camp advocate treatment with the arm flexed to a right angle, and advocate early passive motions. By this treatment it is difficult to prevent the loss of the carrying function, unless particular pains be taken in securing the fragmina in their right position and maintaining the obliquity of the joint-line; but that it can be done, if due care be taken, I have had many examples of in my own practice. The joint ought to be examined frequently, under narcosis, if necessary.

If anchylosis should, nevertheless, occur, it will, at least, give a useful arm, and a moderate diminution of the obtuse angle does not seriously injure the function of the joint. I have treated a large number of these cases, and my results have uniformly been good. My rule is always to examine the patient under narcosis, reduce the displacement, if possible, and then mold an anterior right-angled splint of half-tanned leather, which is applied with lino-bandages. The bend of the splint in the elbow I make oblique, to correspond with the normal obliquity of the joint. The day following I cut a window posteriorly over the joint without disturbing the splint, and satisfy myself that the fragmina are in normal position. If correct, I leave the arm alone for ten or twelve days, and commence then passive motions every two or three days, at first, of course, very lightly and moderately. The splint is removed permanently as soon as there is apparent union (sixteen to twenty days), and the child encouraged to use the arm. In four weeks I have generally managed to discharge the patient with a good result. In cases in which anchylosis has occurred in an almost straight position, operation is absolutely indicated, and no sure diagnosis can be made before the bones are exposed. With proper antiseptic precautions, the operation is devoid of all danger. I prefer in these cases to use Huetter's operation with two lateral incisions, one three inches long, over the posterior surface of the radius and external condyle, and one, one inch long, just anterior to the internal condyle. The operation is done subperiostally, and the lower end of the humerus brought out through the radial wound. We can then decide what damage has been done and how to repair it, be that by removing superfluous callus with the chisel, or resecting the articulating surface of the humerus. I have generally left the ulna and radius intact when the upper epiphysis was removed. It has the advantage that no lateral motion occurs in the joint, but the disadvantage that perfect extension and flexion become impaired, unless we remove the tips of the olecranon and coronoid processes. By removing the articulating surface, we shorten the humerus, at least, one-half inch, and the tips of the olecranon and coronoid processes will come in contact with the humerus above the intercondyloid fossa in extension and flexion.

I have lately operated three cases of anchylosis in a straight position :

Case IX.—Agnes Whitmarsh, 6 years old, of Wellsville, N. Y., injured her left elbow by falling down-stairs, in March, 1892. She entered the Sisters' Hospital in July, 1892, with her left elbow partly anchylosed in an almost straight position.

The olecranon was very prominent, and above the intercondyloid line a large mass of bone was felt in the plica cubiti. Huetter's operation was performed and the humerus brought out through the radial incision, and the following condition found : A T fracture had occurred at the lower end of the humerus. The external condyle and epicondyle were found moved upwards and its articulating surface turned forwards. The trochlea was also found turned forwards. The ulna was dislocated backwards, the coronoid process being found strongly adherent to the posterior intercondyloid fossa. The fossa olecrani was filled with fibrous tissue, which was removed, as was the articulating surface of the humerus. She returned home in four weeks with a useful and excellent arm, but unable to completely extend the arm.

Case X. — Michael Mintugh, 9 years old, of Dunkirk, N. Y., entered the Sisters' Hospital on August 28, 1893. He fell, on the 13th of February, 1893, and struck his left elbow. On examination, the arm was found in almost complete extension and anchylosis. The olecranon was found one-half inch above the condyles of the humerus. The external condyle was very prominent, while the internal was scarcely felt. A large rounded swelling was found in the plica cubiti, evidently connected with the lower epiphysis of the humerus. The arm was strongly adducted in the elbow-joint. The diagnosis was that of unrecognized dislocation, and arthrotomy was done after Huetter's method. A curious deformity was found of the lower end of the humerus. The internal condyle of the humerus had disappeared, and a large external condyle was found. Evidently, a supracondylar fracture had occurred, and the lower fragmen had moved inwards and become attached over the inner condyle, which, therefore, apparently had disappeared. The ulna was dislocated backwards, and the coronoid process was strongly attached to the posterior intercondyloid fossa. The lower epiphysis of the humerus was removed, and the wound sutured without drainage. It healed by first intention. Passive motions were commenced in the second week, and he left the hospital on October 1, 1893, with almost perfect extension and flexion.

Case XI.—Ethel Cotton, 3 years old, of Buffalo, entered the hospital on November 15, 1893, with a deformed arm, the result of a fall four weeks previously. The arm was almost fully extended, and only very small motions were possible. The carrying function had disappeared, the forearm being strongly adducted in the elbow. Arthrotomy of the joint was done after Huetter's method, and a supracondylar fracture found. The lower anterior margin of the upper fragmen formed a prominent ridge anteriorly, and there was here found an abnormal development of callus, which met the coronoid process of the ulna as soon as flexion was begun. The oblique line of the joints had disappeared, the inner end of the lower fragmen having moved upwards, and the joint-line was oblique in the opposite direction, *i. e.*, upwards and inwards. The prominent ridge and callus were removed with a chisel, but the epiphysis was left intact. The wound was sutured without drainage. Immediately after the removal of the ridge the arm could be flexed normally. Nothing could, in my estimation, be done to restore the lost carrying function, as the whole epiphysis had had its periosteum stripped off during the operation. Otherwise, a wedge-shaped osteotomy, with the base outwards, might perhaps have rectified it.

November 28th, arm dressed, and wound found healed by first intention, passive motions commenced, and the patient discharged to her home.

